# Aspirin: Friend or enemy?

**DOI:** 10.1016/j.ijcha.2021.100874

**Published:** 2021-09-21

**Authors:** Wojciech Marlicz, Dafni Charisopoulou, Anastasios Koulaouzidis, George Koulaouzidis

**Affiliations:** a Department of Gastroenterology, Pomeranian Medical University, Szczecin 71-252, Poland; b Amalia Children's Hospital, Radboud University Medical Centre, Nijmegen, Netherlands; c Academic Centre for Congenital Heart Disease, Netherlands; d Department of Social Medicine and Public Health, Pomeranian Medical University, Szczecin, Poland; e Department of Biochemical Sciences, Pomeranian Medical University, Szczecin, Poland

We read with interest the study by Biccler et al. on the public health impact of low-dose aspirin on colorectal cancer (CRC), cardiovascular disease (CVD) and safety in the UK [1]. Based on micro-simulation model data representing the epidemiology in the United Kingdom (UK) for which high-quality data was available, the authors attempt to evaluate the population-level expected benefits and risks of regular use of low-dose aspirin therapy. The model focuses on adults eligible for low-dose aspirin treatment for primary or secondary CVD prevention, with the evidence assessment on the effect of aspirin for CRC prevention. The decrease in both fatal CVD and CRC events was more significant than the increase in catastrophic safety events, and this difference was more pronounced when low-dose aspirin was used. These findings are essential in initiating wide prophylaxis in the era of the Covid-19 pandemic, where environmental risks drive societies towards unhealthy diets and lifestyles.

Significant questions and concerns have been raised after publishing the outcomes Aspirin in Reducing Events in the Elderly (ASPREE) trial [2], [3]. The study aimed to investigate the role of aspirin in the primary prevention of cardiovascular disease, especially in older persons. In this study, 19,114 persons (70 years of age or older or ≥65 years of age among blacks and Hispanics in the United States) free of cardiovascular disease, dementia, or disability were recruited and assigned to receive aspirin or placebo. After a median of 4.7 years of follow-up, higher all-cause mortality was observed among apparently healthy older adults who received daily aspirin than among those who received placebo and was attributed primarily to cancer-related death. Also, low-dose aspirin resulted in a significantly higher risk of major bleeding and did not result in a considerably lower risk of cardiovascular disease than placebo.

An important metanalysis of three large, randomized trials (TIPS-3, HOPE-3, Polyran) with a total of 18,162 participants was published at Lancet [4]. The study aimed to investigate whether aspirin should be included or not on fixed-dose combination treatments (or polypills). The primary outcome was the time to the first occurrence of a composite of cardiovascular death, myocardial infarction, stroke, or arterial revascularisation. Additional outcomes included individual cardiovascular outcomes and death from any cause. Significant reductions in the primary outcome and components were observed in the analyses of fixed-dose combination strategies with and without aspirin, with more substantial reductions for strategies including aspirin. Gastrointestinal bleeding was uncommon but slightly more frequent but not statistically significant in the fixed-dose combination strategy with aspirin group versus control (0.4% *vs* 0.2%, p = 0.15). Similarly, the frequencies of haemorrhagic stroke, fatal bleeding, and peptic ulcer disease were low and did not differ significantly between groups.

The recent study by Biccler et al. and other recent high impacted trials on the effect of aspirin on CVD and CRC risks have high translational values into the daily management of CVD. Results like that could be easily translated and implemented into daily protocols of cardiovascular disease management, tempting healthcare practitioners to advocate dietary changes combined with polypill pharmacotherapy as evidence-based medicine among patients without cardiovascular disease with intermediate cardiovascular risk. Moreover, in the pandemic era with an increase in non-communicable diseases, healthcare professionals are tempted to advocate lifestyle and dietary changes, frequently combined with aspirin administration in their patients, including those without CVD and with intermediate cardiovascular or cancer risk.

However, several key issues remain to be addressed before announcing the full safety of these approaches:

1. the complexity of food-drug-microbiome interactions and potential ability of certain foods combined with polypill and aspirin to alter the gut microbiome and intestinal mucosa triggering yet undesired or unknown metabolic effects
2. higher all-cause mortality attributed to cancer deaths in healthy older adults receiving daily aspirin
3. association between the NSAIDs use and various dietary regimens with increased risk of Clostridium Difficile diarrhoea, at least in individuals at intermediate cardiovascular risk in the Covid-19 era

In conclusion, Biccler et al. study is a forward stride in developing a safe approach to minimize further CVD and CRC risks. However, considering recent findings, full implementation of low-dose aspirin supplementation in selected groups of individuals requires multidisciplinary efforts supported by innovative research.

## Declaration of Competing Interest

The authors declare that they have no known competing financial interests or personal relationships that could have appeared to influence the work reported in this paper.
